# GC/MS-based quantitative analysis of sulfide ion in whole blood using ethenesulfonyl fluoride as a derivatization reagent

**DOI:** 10.1007/s11419-025-00712-9

**Published:** 2025-02-10

**Authors:** Ryosuke Shiraki, Shin Ogawa, Kengo Wakigawa, Hidehiko Okazaki, Akinaga Gohda, Takeshi Mori, Yoshiki Katayama

**Affiliations:** 1 Graduate School of Systems Life Sciences, Kyushu University, 744 Motooka, Nishi-ku, Fukuoka, 819-0395 Japan; 2 Department of Applied Chemistry, Faculty of Engineering, Kyushu University, 744 Motooka, Nishi-ku, Fukuoka, 819-0395 Japan; 3 Center for Future Chemistry, Kyushu University, 744 Motooka, Nishi-ku, Fukuoka, 819-0395 Japan; 4 International Research Center for Molecular Systems, Kyushu University, 744 Motooka, Nishi-ku, Fukuoka, 819-0395 Japan; 5 Center for Advanced Medical Innovation, Kyushu University, 3-1-1 Maidashi, Higashi-ku, Fukuoka, 812-8532 Japan; 6 Forensic Science Laboratory, Fukuoka Prefectural Police Headquarters, 7-7 Higashikoen, Hakata-ku, Fukuoka, 812-8576 Japan

**Keywords:** Hydrogen sulfide, Sulfide ion, Gas chromatography/mass spectrometry, Ethenesulfonyl fluoride, Derivatization

## Abstract

**Purpose:**

Identification and quantification of sulfide ion in biological samples are required in forensic purpose. Gas chromatography-mass spectrometry (GC/MS) has been used for the analysis of sulfide ion by using derivatization reagents. However, conventional derivatization reagents require special attention for derivatization. To simplify the derivatization protocol, we examined ethenesulfonyl fluoride (ESF) as a derivatizing reagent of sulfide ion.

**Methods:**

To 100 μL of whole blood sample containing sulfide ion, 100 μL of boric acid buffer (pH 8.0), 100 μL of acetone solution containing internal standard, 100 μL of acetone solution containing 600 mM concentration of ESF, and 100 μL of hexane were added in a 1.5-mL plastic tube. The mixture was vortexed at room temperature, the tubes were centrifuged, and the organic layer was injected into the GC/MS.

**Results:**

ESF exhibited higher reactivity toward sulfide ion than interfering compounds present in whole blood, allowing for selective derivatization. With the optimized protocol, the detection limit for sulfide ion was 0.01 μg/mL. The calibration curve showed good linearity (*R*^2^ = 0.9999) in the range of 0.05–10.0 μg/mL, and the precision (% relative standard deviation) and the accuracy (% bias) were within ± 10% (intra- and inter-day).

**Conclusion:**

This GC/MS-based method is a valuable tool for forensic investigations and various analytical fields, offering reliable quantification of sulfide ion in whole blood.

**Supplementary Information:**

The online version contains supplementary material available at 10.1007/s11419-025-00712-9.

## Introduction

Hydrogen sulfide (H_2_S) is a gas with a rotten egg smell and is naturally emitted from volcanic regions, hot springs, rotting organic matter, and paper mills [1–5]. H_2_S has beneficial effects at low concentrations, including neural and cellular protection, antiepileptic effects, and potential in diabetes treatment [6–8]. However, inhalation at high concentrations is toxic, leading to frequent incidents of poisoning in areas where H_2_S is generated [4, 9]. In the poisoning cases, the poisoning typically occurs through the ingestion of pesticides containing sulfides, or by inhaling H_2_S gas produced by mixing such pesticides with acidic cleaning agents [10, 11]. The blood concentration of sulfide ion is reported to be below 0.05 μg/mL, whereas in cases of fatal poisoning due to H_2_S exposure, it has been reported to range from 0.32 to 2.56 μg/mL [4, 12, 13]. H_2_S is an important analyte in forensic chemistry for investigating the causes of incidents and accidents. However, H_2_S is decomposed by oxygen in the blood [14, 15], thus, rapid methods are required for the detection of H_2_S in biological samples.

Although colorimetric methods [16–18], gas chromatography [19–23], and liquid chromatography [24, 25] are rapid detection methods of H_2_S, they lack specificity to identify the sulfide ion. Mass detection is generally specific, but the low mass of H_2_S make it difficult to identify. To improve the specificity of mass identification of sulfide ion, its derivatization is useful because of the increase in molecular weight and specific pattern of the mass of fragmented compounds. Pentafluorobenzyl bromide (PFBBr) has been used for the derivatization of sulfide ion by gas chromatography-mass spectrometry (GC/MS). However, its tear-gas-like irritating nature requires careful handling and the hydrophobic nature of this reagent increases the deviation of the result due to the necessity of using a surfactant [26, 27]. Monobromobimane has been used for the derivatization of sulfide ion in liquid chromatography-mass spectrometry. However, the procedure is time-consuming, and resulting derivative is light-sensitive and decomposes upon exposure, requiring it to be handled in the dark [25, 28].

**Fig. 1:**
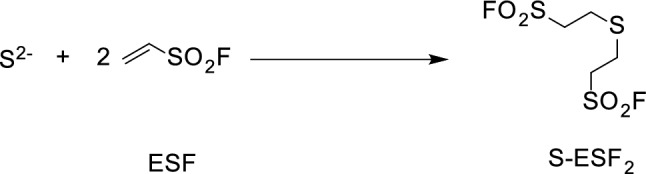
The derivatization reaction of sulfide ion by ethenesulfonyl fluoride (ESF)

Here, we propose ethenesulfonyl fluoride (ESF) as a novel derivatizing reagent for the analysis of sulfide ion present in whole blood using GC/MS. ESF is a highly reactive Michael acceptor due to the electron-deficient α,β-unsaturated bond it possesses [29, 30]. Notably, ESF exhibits higher reactivity toward sulfur compounds than water and aliphatic alcohols [31–34]. We have previously reported a method for the derivatization of ammonia in water using ESF, followed by analysis by GC/MS, which demonstrated that ESF is an effective derivatization reagent for the analysis of water-soluble compounds [35]. Thus, ESF will enable the derivatization of sulfide ion in aqueous and alcoholic solutions. We found here that ESF rapidly reacted with sulfide ion in whole blood and formed a suitable di-ESF derivative (S-ESF_2_) that can be separated and detected using GC/MS (Fig. 1).

## Materials and methods

### Materials

Sodium sulfide (FUJIFILM Wako Pure Chemical Corp., Osaka, Japan) was dissolved in water and titrated [36]. The titrated solution was prepared at a concentration of 1000 μg/mL to create a standard sulfide ion solution. This standard sulfide ion solution was diluted with water to the desired concentrations and added to the whole blood as samples. The derivatization reagent was prepared by diluting ESF (Tokyo Chemical Industry Co., Ltd., Tokyo, Japan) to a concentration of 5% by volume with acetone. Phenanthrene-*d*_10_ (FUJIFILM Wako Pure Chemical Corp.) was used as the internal standard (IS) and dissolved in acetone to a concentration of 1.0 μg/mL. To prevent the decomposition of H_2_S due to dissolved oxygen [14, 15], the water and organic solvents used in the experiment were bubbled with nitrogen to remove oxygen [26]. The purified water used in this study was obtained from a Milli-Q Integral 5 system (Merck Millipore, Billerica, MA, USA). The commercially available human whole blood was purchased (Cosmo Bio Corp., Tokyo, Japan).

The reagents used for headspace-gas chromatography-mass spectrometry (HS-GC/MS) analysis included phosphoric acid (FUJIFILM Wako Pure Chemical Corp.). For pentafluorobenzyl (PFB) derivatization GC/MS analysis, the reagents included tetradecyldimethylbenzylammonium chloride (Tokyo Chemical Industry Co., Ltd.), pentafluorobenzyl bromide (Sigma-Aldrich, St. Louis, MO, USA), 1,3,5-tribromobenzene (FUJIFILM Wako Pure Chemical Corp.), and potassium dihydrogen phosphate (FUJIFILM Wako Pure Chemical Corp.). All other chemicals used were analytical grade and commercially available.

### ESF derivatization and extraction procedure

First, 100 μL of a whole blood sample containing sulfide ion was added to a 1.5-mL plastic tube. Next, 100 μL of borate buffer solution (pH 8.0), 100 μL of acetone solution containing IS, 100 μL of acetone solution containing ESF at a concentration of 600 mM, and 100 μL of hexane were added to the tube. The mixture was vortexed at room temperature for 1 min. The tube was then centrifuged at 2000×*g* for 1 min. The organic layer was placed in a separate glass tube, and 1.0 μL of this solution was injected into the GC/MS.

### HS-GC/MS analysis procedure

The analysis using HS-GC/MS was conducted with reference to prior reports [27]. In a glass vial with a volume of 20-mL, 500 μL of a whole blood sample containing sulfide ion and 500 μL of an aqueous solution containing 0.01 vol% of methanol as IS were added and mixed. To this solution, 1 mL of acetone was added and vortexed for 5 s. Subsequently, 500 μL of phosphoric acid was added, the vial was quickly sealed, and it was vortexed for 5 s. Afterward, the analysis was performed using HS-GC/MS (Fig. S1).

### PFB derivatization and extraction procedure

The method for derivatization GC/MS using PFBBr was adapted from previously reported techniques [4]. In a 10-mL test tube, 0.8 mL of 5 mmol/L tetradecyldimethylbenzylammonium chloride aqueous solution, 0.5 mL of 20 mmol/L PFBBr in ethyl acetate, and 2.0 mL of 1.0 μmol/L 1,3,5-tribromobenzene in ethyl acetate as an IS were added. To this solution, 200 μL of whole blood sample containing sulfide ion was added and then vortexed for 1 min. Then, 0.10 g of potassium dihydrogen phosphate was added, and the solution was vortexed for another 10 s. The test tube was then centrifuged at 2000×*g* for 1 min. The organic layer was transferred to a separate glass container, and 1.0 μL of it was analyzed using GC/MS (Fig. S2).

### GC/MS analysis

GC/MS determination was performed on a GCMS-QP2020 NX (Shimadzu, Kyoto, Japan) equipped with a DB-5 MS capillary column (30 m × 0.25 mm *i.d.*, 0.25 μm film thickness, Agilent Technologies, Santa Clara, CA). The GC oven temperature was held at 80 °C for 1 min and increased to 320 °C at 20 °C/min (held for 2 min). The injection mode was splitless and the carrier gas was helium at a flow rate of 20.2 mL/min. The injection volume was 1.0 μL. The MS interface temperature was 250 °C and the ion source temperature was 250 °C. The system was operated with a filament current of 150 μA, an electron energy of 70 eV, and in electron ionization (EI) mode. The derivative was identified in full scan mode, and data were acquired from *m/z* 30 to 300. Quantitation was performed in selective ion monitoring (SIM) mode, monitoring ions at *m/z* 86, 170, and 254 for S-ESF_2_ and *m/z* 160, and 188 for the IS (the quantitation ions are underlined).

The analytical conditions for derivatization GC/MS using PFBBr were as follows. The GC oven temperature was held at 100 °C for 2 min and increased to 290 °C at 10 °C/min (held for 5 min). The injection mode was splitless and the carrier gas was helium at a flow rate of 10.7 mL/min. The injection volume was 1.0 μL. The derivative was identified in full scan mode, and data were acquired from *m/z* 30 to 500. Quantitation was performed in SIM mode, monitoring ions at *m/z* 181, and 394 for S-PFB_2_ and *m/z* 235, and 314 for the IS (the quantitation ions are underlined).

### HS-GC/MS analysis

HS-GC/MS determination was performed on a GCMS-QP2020 NX instrument (Shimadzu) equipped with a Pora PLOT Q-HT capillary column (25 m × 0.32 mm *i.d.*, 10 μm film thickness, Agilent Technologies). An HS-20 headspace autosampler (Shimadzu) was used. The oven temperature for the headspace autosampler was set at 60 °C, the sample line temperature at 100 °C, and the transfer line temperature at 150 °C. The vial equilibration time was 10 min, helium was used as the vial pressurization gas with a vial pressurization time of 0.5 min, and a pressurization equilibration time of 0.1 min. The loading time was 0.5 min with no loading equilibration time, the injection time was 0.5 min, and the needle flush time was 5 min. The GC oven temperature was initially held at 40 °C for 1.5 min, then increased to 250 °C at a rate of 25 °C/min (held for 1 min). The injection mode was set to split (split ratio 1:5), and helium was used as the carrier gas with a flow rate of 11.6 mL/min. The MS interface temperature was 230 °C, and the ion source temperature was 200 °C. The system was operated with a filament current of 150 μA, an electron energy of 70 eV, and in EI mode. Quantitation was performed in SIM mode, monitoring ions at *m/z* 34 and 33 for H_2_S, and *m/z* 31 and 29 for the IS (quantification ions are underlined).

### **Comparison of S-ESF**_**2**_**production in whole blood relative to water**

The production of S-ESF_2_ in the above ESF derivatization procedure was compared between whole blood and water matrices at sulfide ion concentrations of 0.1, 1.0, and 8.0 µg/mL (*n* = 3).

### Validation procedures for whole blood samples

Five blank whole blood samples were analyzed five times each to confirm the absence of interfering peaks.

Standard solutions and blank whole blood were mixed to prepare samples containing 0.05, 0.25, 0.5, 2.5, 5.0, and 10 μg/mL of sulfide ion. The samples were extracted using the method described above and analyzed by GC/MS. The limit of detection (LOD) was defined as a signal-to-noise (S/N) ratio greater than 3, and the limit of quantification (LOQ) was defined as an S/N ratio of 10. Quality control samples were independently prepared at 0.05, 0.1, 1.0, and 8.0 μg/mL and quantified using the above calibration curve. Intra-day precision and accuracy were assessed based on the calculated concentrations of five samples spiked at four levels, extracted, and analyzed on the same day. Inter-day precision and accuracy were assessed based on the average concentrations measured at four levels over three days [37].

The dilution integrity was evaluated by diluting samples corresponding to 25 and 50 µg/mL concentrations to 5.0 µg/mL using blank whole blood (*n* = 5).

## Results

### Optimization of protocol

**Fig. 2:**
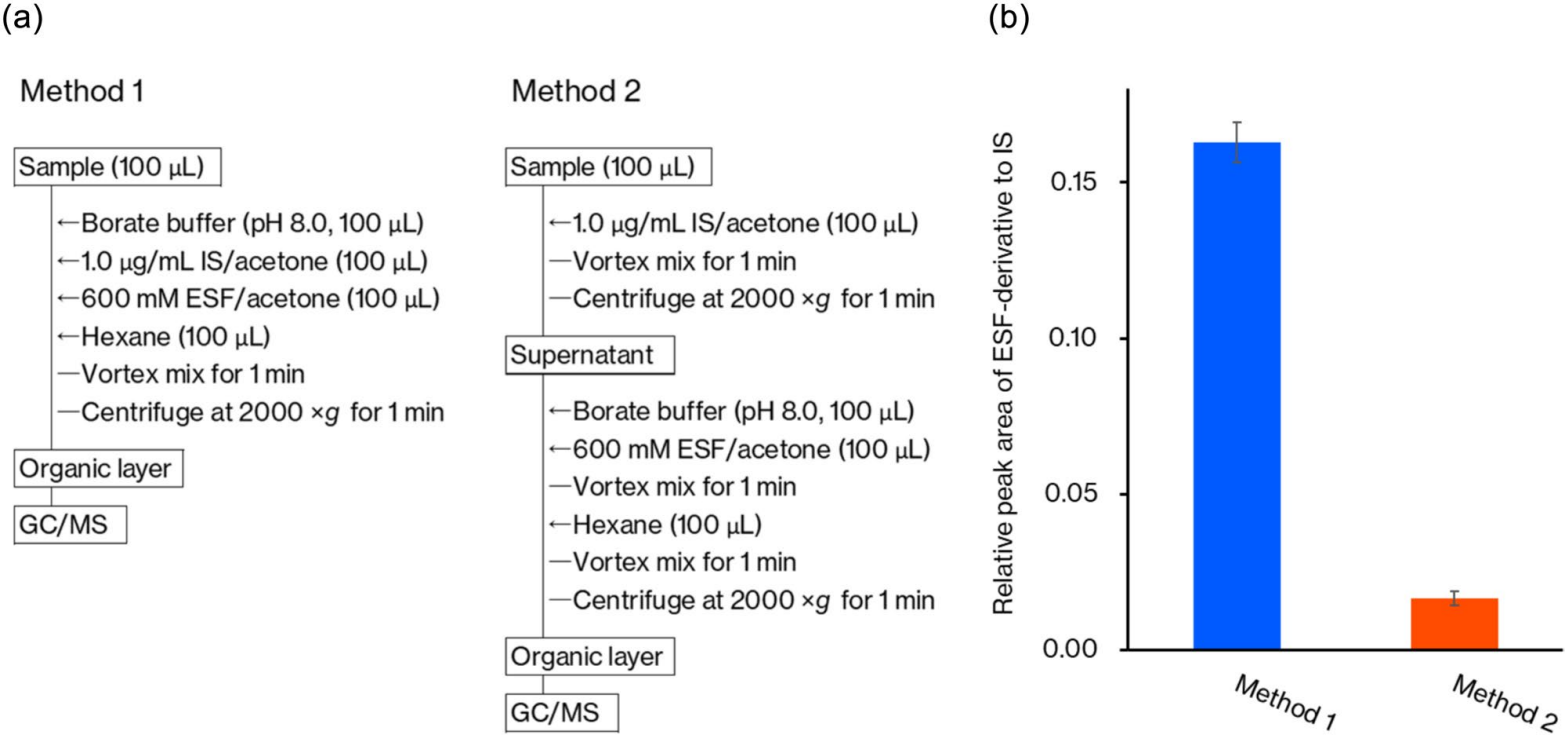
Comparison of derivatization Methods 1 and 2 for sulfide ion analysis in whole blood. Procedures of the two methods (**a**) and a comparison of the derivatization efficacy (**b**). Each operation was performed three times, and the mean value is presented with a standard deviation

**Fig. 3:**
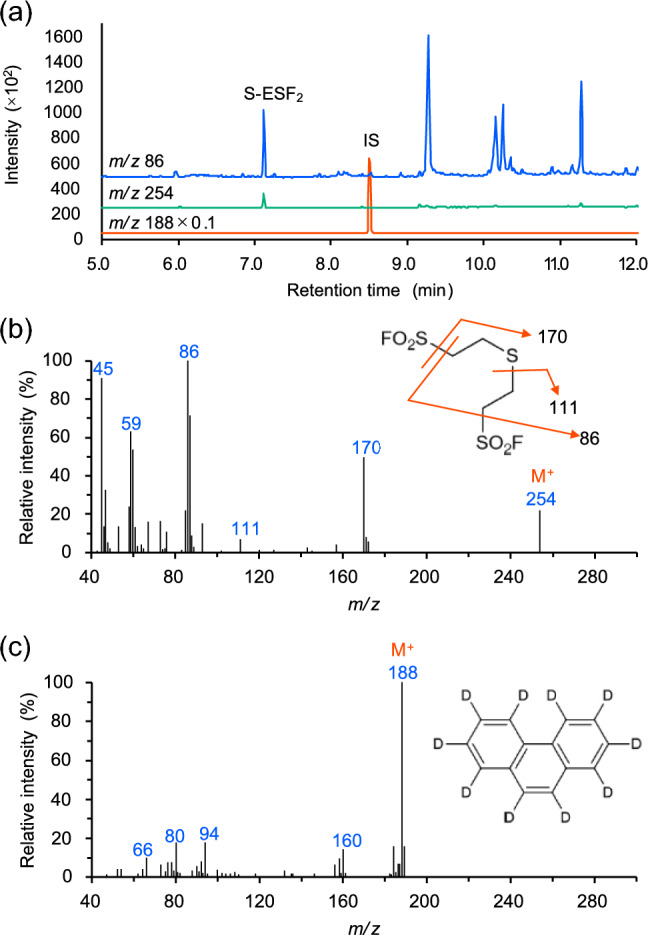
Extracted ion chromatograms of the ESF-derivatized sulfide ion (**a**). Mass spectra of S-ESF_2_ (**b**) and the internal standard (IS), phenanthrene-*d*_10_ (**c**). Components with *m/z* 86, 254, and 188 detected in panel A were assignable to fragment ion of S-ESF_2_, molecular ion of S-ESF_2_, and molecular ion of IS, respectively

We examined the derivatization of sulfide ion spiked in whole blood using ESF. The derivatization reaction was performed using Method 1 shown in Fig. 2a. A whole blood sample (100 μL) spiked with sulfide ion (1.0 μg/mL, 0.03 mM) was mixed with 100 μL of borate buffer solution (pH 8.0), 100 μL of acetone solution of ESF (600 mM), and 100 μL of acetone solution of phenanthrene-*d*_10_ (1.0 μg/mL) used as an IS. To the mixture, 100 μL of hexane was added. Then, the resulting dispersion was vortexed at room temperature for 1 min. After the reaction, the dispersion was centrifuged for precipitation of protein fractions to separate organic and aqueous phases, then the organic hexane layer was analyzed using a low-polarity GC column. We successfully detected S-ESF_2_ in the extracted ion chromatograms (Fig. 3a). In addition to the molecular ion peak at *m/z* 254, fragment ions at *m/z* 86, 111, and 170 were observed (Fig. 3b). The peaks at *m/z* 86 and 170 correspond to the fragments with the loss of double and single -SO_2_F groups, respectively. The peak at *m/z* 111 indicates the loss of the -CH_2_CH_2_SO_2_F group. The peak at *m/z* 86 was the base peak. It is notable that the mono-ESF derivative was not detected in the mass chromatogram even with a very short reaction time, 1 min. This suggests that the mono-ESF derivative rapidly reacts with ESF to form S-ESF_2_. When five blank whole blood samples were analyzed to confirm the specificity of the method, no peaks that interfered with the derivatives were observed.

We evaluated the stability of S-ESF_2_ extracted into the hexane phase during storage at room temperature (Fig. S3). No loss of S-ESF_2_ was observed for at least 36 h. Considering the easily oxidized property and high volatility of sulfide ion, the high stability of S-ESF_2_ is advantageous for practical analysis.

In Method 1, the derivatization of sulfide ion and protein precipitation occurred simultaneously. We compared the efficacy of derivatization of sulfide ion with Method 2 in which the derivatization was performed on the supernatant after precipitation of proteins in the blood (Fig. 2). The efficacy of derivatization was much lower in Method 2, which may be due to the distribution of sulfide ion into the precipitated proteins.

**Fig. 4:**
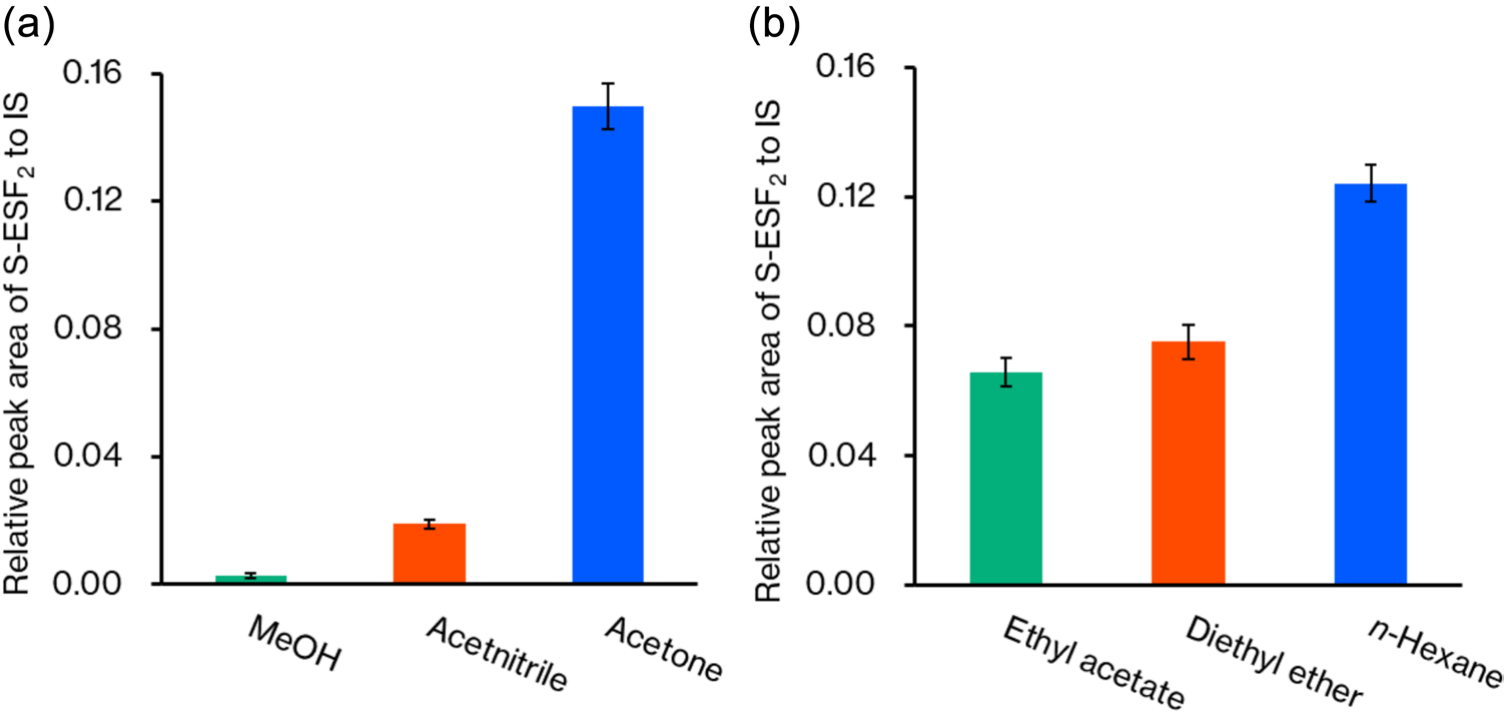
Effect of different protein precipitation solvents (**a**) and different extraction solvents for the derivatized samples (**b**). Each operation was performed three times, and the mean value is presented with a standard deviation

Method 1 was performed by optimizing the following factors: protein precipitation solvent, extraction solvent, reaction time, and reaction pH. Figure 4a shows the effect of protein precipitation solvent. Acetone provided the highest concentration of S-ESF_2_ although its protein removal efficacy is not the highest among the three examined organic solvents (the protein removal efficacy: methanol < acetone < acetonitrile [38]). Thus, the high removal efficacy of acetonitrile may result in coprecipitation of sulfide ion with proteins, leading to the low efficacy of derivatization. Figure 4b shows the effect of extraction solvents on the recovery of S-ESF_2_. Hexane showed the highest extraction efficiency, indicating that a nonpolar solvent is more suitable for the extraction of S-ESF_2_.

**Fig. 5:**
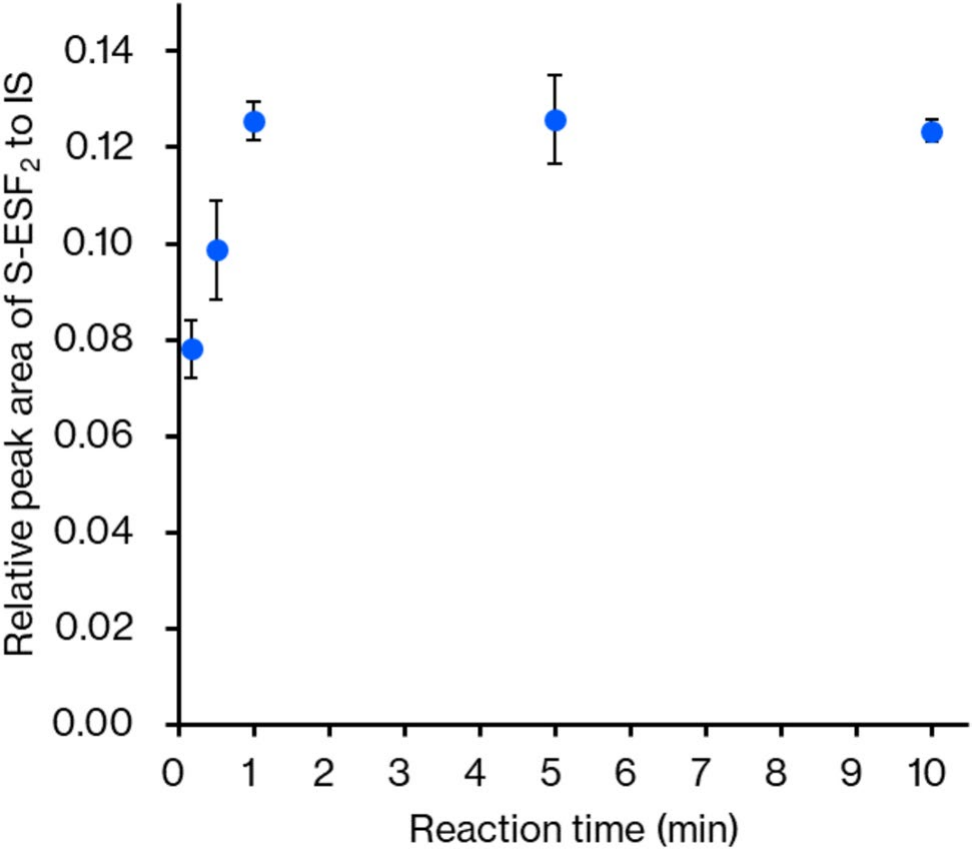
Effect of the reaction time on sulfide ion derivatization. Each experiment was performed three times, and the mean value is presented with a standard deviation

**Fig. 6:**
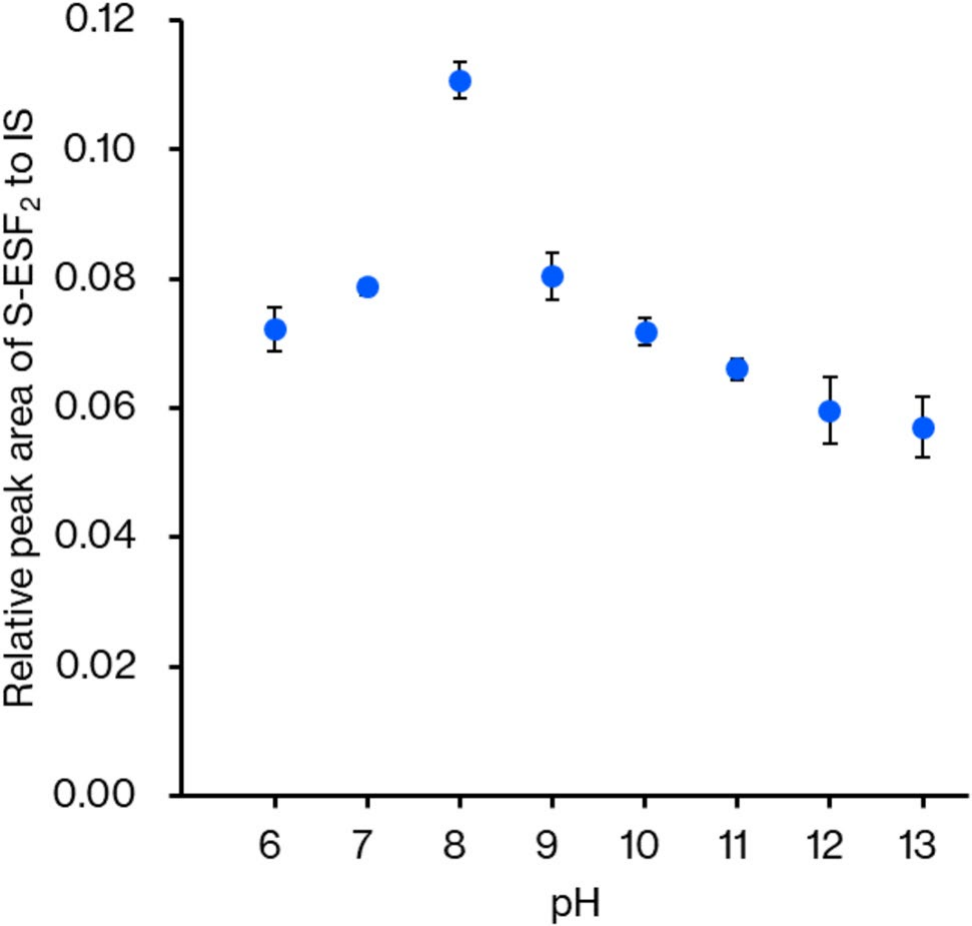
Effect of the buffer solution pH on derivatization. Each operation was performed three times, and the mean value is presented with a standard deviation

Figure 5 shows the effect of the reaction time of derivatization on the recovered amount of S-ESF_2_ derivative. The amount of S-ESF_2_ had almost plateaued at 1 min, proving the rapid reaction of S-ESF_2_ formation. Figure 6 shows the effect of the buffer pH on the derivatization of sulfide ion with ESF. Because the yield of S-ESF_2_ was found to be highest at pH 8, we set pH 8 as the optimal pH for the derivatization. The lower yields at a lower pH (pH < 8) will result from the low reactivity in derivatization due to the protonation of sulfide ion at low pH (p*K*_a1_ of H_2_S is 6.9 [39]). The lower yields at higher pH (pH > 8) may be caused by the reaction of ESF with lysine residues of proteins in the blood, which interfere with the derivatization of sulfide ion.

The production of S-ESF_2_ was compared between whole blood and water matrices using three different concentrations of sulfide ion. The results showed that the production of S-ESF_2_ in whole blood was 71–77% of that in water (Table S1). Thus, for quantifying sulfide ion in whole blood, a calibration curve was prepared with whole blood samples as the matrix.

### Analytical ability

**Fig. 7:**
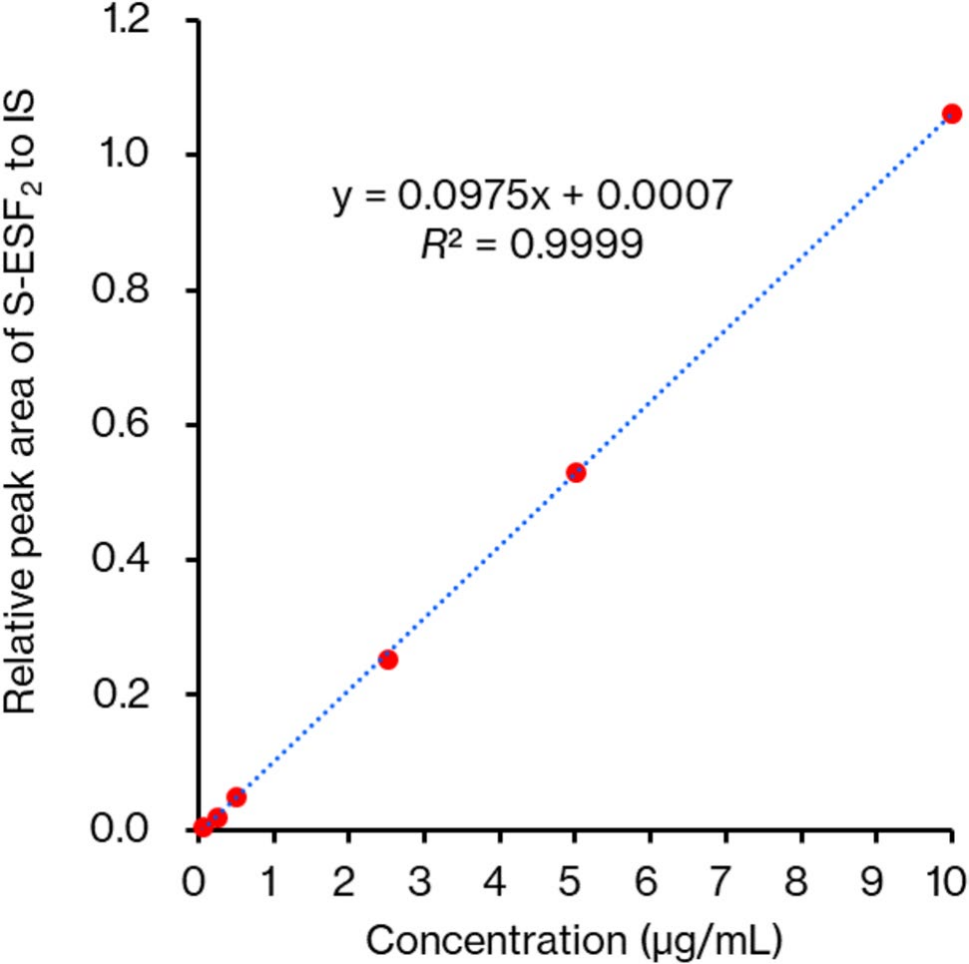
Calibration curve showing the peak areas for S-ESF_2_ generated from sulfide ion concentrations ranging from 0.05 to 10.0 μg/mL

A calibration curve was prepared using solutions with sulfide ion concentrations ranging from 0.05 to 10.0 μg/mL (0.0015–0.31 mM) (Fig. 7). The blank whole blood sample used in these experiments was also analyzed, and no sulfide ion were detected. The linearity of the calibration curve was evaluated based on the relationship between the peak area of S-ESF_2_ and the sulfide ion concentration. The area ratio of the base peak of S-ESF_2_ at *m/z* 86 to the base peak of the IS at *m/z* 188 was plotted against the sulfide ion concentration. Linear regression analysis using the weighting factor of 1/*x* showed good linearity (*R*^2^ = 0.9999).

To assess the precision and accuracy of the method, we analyzed whole blood samples adjusted to four concentrations (0.05, 0.1, 1.0, and 8.0 μg/mL) five times each, and performed the analysis for three days (Table 1). The LOD based on a S/N ratio of 3 was 0.01 μg/mL, and the LOQ based on a S/N ratio of 10 was 0.05 μg/mL. The results showed that the method was very robust and highly reproducible.

**Table 1:** Intra- and inter-day precision and accuracy

Nominal concentration (μg/mL)	Intra-day (*n* = 5)			Inter-day (*n* = 3)		
	Mean ± SD ^a^	Precision (% RSD) ^b^	Accuracy (% bias) ^c^	Mean ± SD ^a^	Precision (% RSD) ^b^	Accuracy (% bias) ^c^
0.05	0.05 ± 0.001	1.9	3.1	0.05 ± 0.001	2.1	2.1
0.1	0.10 ± 0.003	3.4	− 2.8	0.10 ± 0.003	3.2	1.6
1.0	0.99 ± 0.034	3.4	− 1.1	0.99 ± 0.005	0.5	− 1.5
8.0	8.30 ± 0.119	1.4	3.7	8.08 ± 0.174	2.2	1.0

^a^Standard deviation

^b^Precision (% relative standard deviation) was defined as (standard deviation/mean peak area ratio to the IS) × 100

^c^Accuracy (% bias) was defined as [(measured concentration − nominal concentration)/nominal concentration] × 100

To address concentrations exceeding the calibration range, whole blood samples were diluted fivefold and tenfold and evaluated. As a result, the precision for fivefold and tenfold dilutions was 2.9% and 3.3%, respectively, while the accuracy for fivefold and tenfold dilutions was − 2.1% and − 8.3%, respectively (Table 2).

**Table 2:** Dilution integrity

Concentration before dilution (μg/mL)	Dilution factors	Concentration after dilution (μg/mL)	Precision (% RSD)^a^	Accuracy (% bias)^b^
25	5	5	2.9	− 2.1
50	10	5	3.3	− 8.3

^a^Precision (% relative standard deviation) was defined as (standard deviation/mean peak area ratio to the IS) × 100 (*n* = 5)

^b^Accuracy (% bias) was defined as [(measured concentration − nominal concentration)/nominal concentration] × 100 (*n* = 5)

### Comparison with conventional methods

The analytical ability of our method was compared with HS-GC/MS [27], which directly detects H_2_S. The whole blood samples spiked with four concentrations of sulfide ion were analyzed three times by each method (Table 3).

**Table 3:** Results of sulfide ion quantification in simulated samples

Nominal concentration (μg/mL)	HS-GC/MS^a^		Proposed method	
	Mean ± SD^b^	Precision (% RSD)^c^	Mean ± SD^b^	Precision (% RSD)^c^
0.5	0.54 ± 0.066	12.1	0.52 ± 0.010	1.9
1.0	1.03 ± 0.133	12.9	1.01 ± 0.018	1.8
5.0	5.25 ± 0.210	4.0	5.29 ± 0.092	1.7
8.0	8.29 ± 0.411	5.0	8.09 ± 0.104	1.3

^a^Values measured by the established HS-GC/MS method [27]

^b^Standard deviation (*n* = 3)

^c^Precision (% relative standard deviation) was defined as (standard deviation/mean peak area ratio to the IS) × 100 (*n* = 3)

In the analysis of sulfide ion in blood, we compared the conventional method with PFBBr as the derivatization reagent [4], to the proposed method (Table 4).

**Table 4:** Comparison of statistical variability with PFB derivatization methods

Nominal concentration (μg/mL)	PFB derivatization^a^		Proposed method	
	Mean ± SD ^b^	Precision (% RSD) ^c^	Mean ± SD^b^	Precision (% RSD)^c^
0.05	0.05 ± 0.008	31.0	0.05 ± 0.001	1.9
0.1	0.08 ± 0.012	20.5	0.10 ± 0.003	3.4
1.0	0.80 ± 0.053	6.9	0.99 ± 0.034	3.4
8.0	8.89 ± 0.443	5.0	8.30 ± 0.119	1.4

^a^Values measured by the established PFB derivatization method [4]

^b^Standard deviation (*n* = 5)

^c^Precision (% relative standard deviation) was defined as (standard deviation/mean peak area ratio to the IS) × 100 (*n* = 5)

## Discussion

### Optimized method

It has been reported that H_2_S in blood exists in three forms: free, reversibly bound, and bound to the blood cells [15, 40]. Conventional analytical methods using derivatization target the free or reversibly bound forms of H_2_S [28]. The protocol here follows that of these methods (i.e., a derivatization reagent, a deproteinization solvent, and an extraction solvent) are simultaneously added to whole blood samples. This protocol allows derivatization to occur concurrently with the precipitation of blood cells by the organic solvent, enabling efficient extraction (Fig. 2).

### Comparison of the present method with conventional methods

To verify the validity of the optimized method, we compared the conventional methods (i.e., HS-GC/MS and PFB derivatization) for sulfide ion analysis. The HS-GC/MS analysis was conducted following the reported protocol [27]. Table 3 summarizes the analytical results of each method. An excellent correlation coefficient was obtained between the two methods (*R*^2^ = 0.9995). Because HS-GC/MS directly detects H_2_S, it lacks distinctive mass spectrum features (Fig. S1), leading to low specificity. In contrast, our method derivatizes sulfide ion, enabling its identification using characteristic fragment ions (Fig. 3b). In addition, the HS-GC/MS method has a high LOQ, it is not suitable for analyzing the trace amount of sulfide ion.

The PFB derivatization analysis was conducted following the reported protocol [4]. It has been pointed out that the derivatization GC/MS method using PFBBr has issues with the precision of quantitative values [26, 27]. Therefore, we compared the relative standard deviation (RSD) at the same concentration for both methods and conducted validation. As shown in Table 4, the RSD was larger for the PFB derivatization method, especially near the lower LOQ. The variability of the PFB derivatization was considered to be attributed to the heterogeneous conditions of the reaction mixture, which used surfactants as phase transfer catalysts. Our ESF derivatization method carried out under homogeneous conditions using aqueous acetone as the solvent, is less likely to cause variability.

## Conclusion

We have proposed ESF as a derivatization reagent for the highly specific GC/MS analysis of sulfide ion in whole blood based on the characteristic fragment ions. ESF rapidly and selectively derivatized sulfide ion without interference from the abundant proteins in whole blood. Compared with a conventional derivatization method using PFBBr, ESF enabled more reproducible derivatization. The high specificity and reproducibility will make our method a valuable technique not only in forensic science but also in various fields of sulfide ion analysis.

## Supplementary Information

Below is the link to the electronic supplementary material.

Supplementary file 1 (DOCX 181 KB)
