# Supplementary material for: GC/MS-based quantitative analysis of sulfide ion in whole blood using ethenesulfonyl fluoride as a derivatization reagent
Source: Forensic Toxicol. 2025 Feb 10;43(2):226–34. doi: 10.1007/s11419-025-00712-9 (PMC12241268; doi:10.1007/s11419-025-00712-9)
Supplement: Supplementary file 1 — (DOCX 181 KB) [file 11419_2025_712_MOESM1_ESM.docx]

**Supporting information**

GC/MS-based quantitative analysis of sulfide ion in whole blood using ethenesulfonyl fluoride as a derivatization reagent

Ryosuke Shiraki^*, a, f^, Shin Ogawa^f^, Kengo Wakigawa^f^, Hidehiko Okazaki^f^, Akinaga Gohda^f^, Takeshi Mori^*, a, b, c^, Yoshiki Katayama^*, a, b, c, d, e^

^a^ Graduate School of Systems Life Sciences, Kyushu University, 744 Motooka, Nishi-ku, Fukuoka 819-0395, Japan

^b^ Department of Applied Chemistry, Faculty of Engineering, Kyushu University, 744 Motooka, Nishi-ku, Fukuoka 819-0395, Japan

^c^ Center for future chemistry, Kyushu University, 744 Motooka, Nishi-ku, Fukuoka 819-0395, Japan

^d^ International Research Center for Molecular Systems, Kyushu University, 744 Motooka, Nishi-ku, Fukuoka 819-0395, Japan

^e^ Center for Advanced Medical Innovation, Kyushu University, 3-1-1 Maidashi, Higashi-ku, Fukuoka 812-8532, Japan

^f^ Forensic Science Laboratory, Fukuoka Prefectural Police Headquarters, 7-7 Higashikoen, Hakata-ku, Fukuoka 812-8576, Japan

* Corresponding author at: Forensic Science Laboratory, Fukuoka Prefectural Police Headquarters, 7-7 Higashikoen, Hakata-ku, Fukuoka 812-8576, Japan and Kyushu University, Graduate School of Systems Life Sciences, 744 Motooka, Nishi-ku, Fukuoka, 819-0395 Japan. Tel./fax: +81 92 802 2850.

E-mail address: s.ryosuke.8975@kyudai.jp (R.S.); mori.takeshi.880@m.kyushu-u.ac.jp (T.M.); katayama.yoshiki.958@m.kyushu-u.ac.jp (Y. K.).


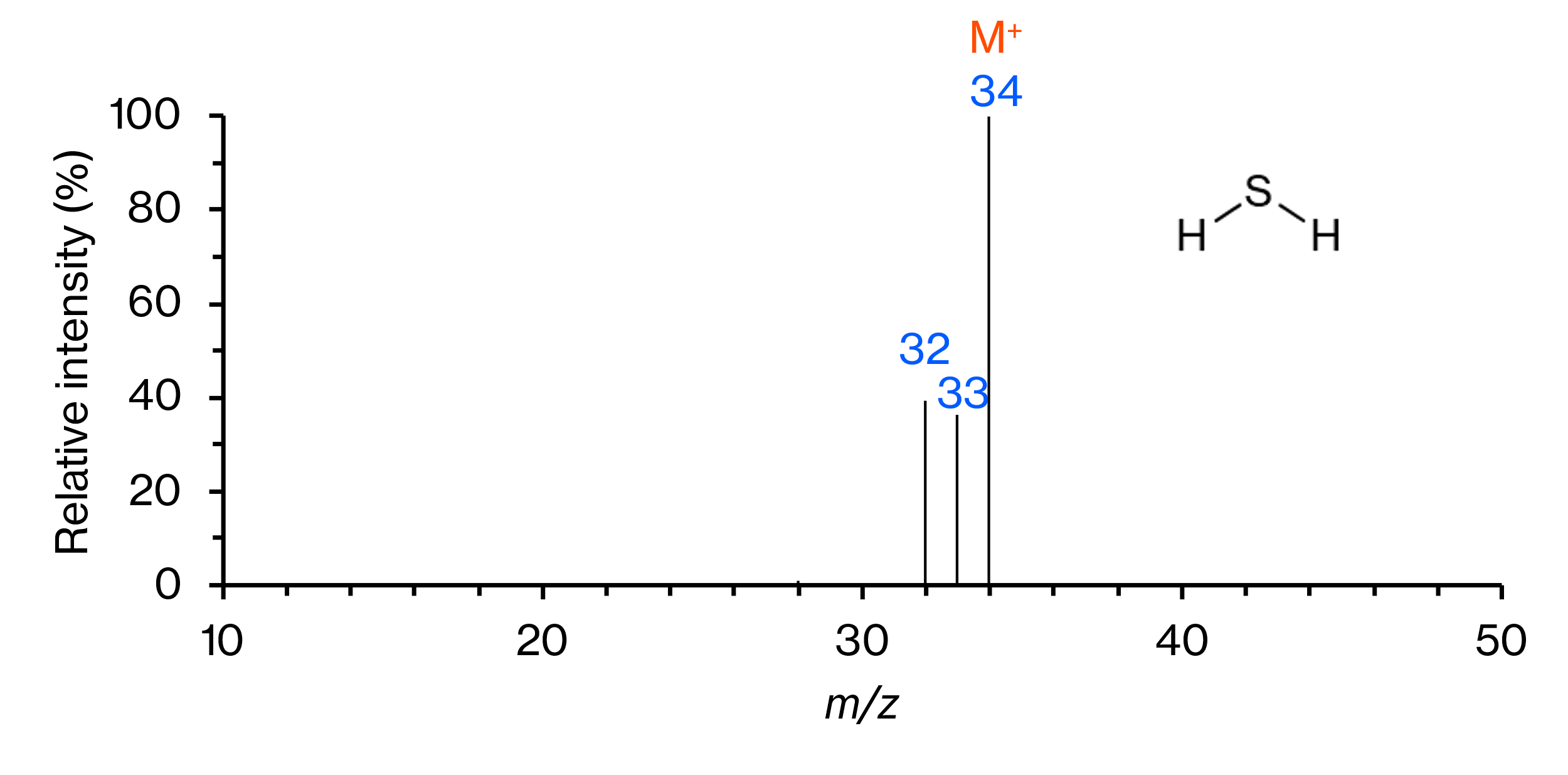


Fig. S1 Mass spectra of hydrogen sulfide. The molecular ion peak at *m/z* 34 was observed for hydrogen sulfide


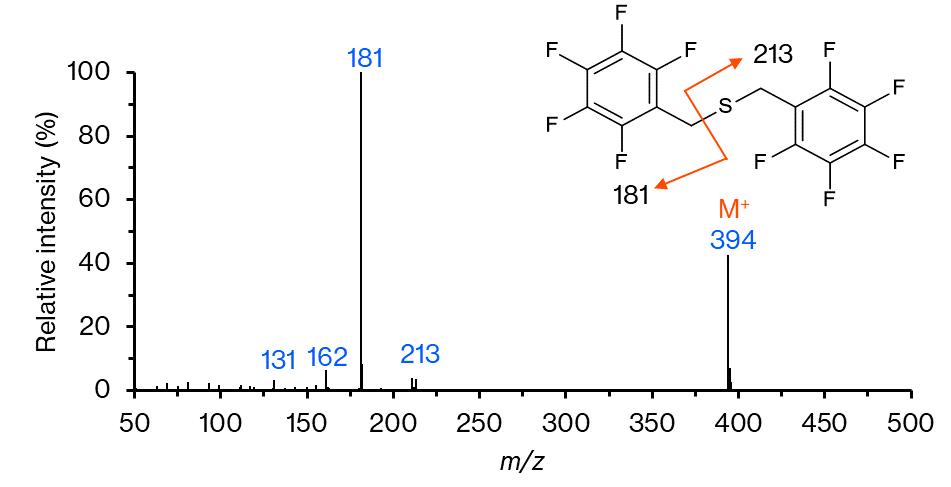


Fig. S2 Mass spectra of S-PFB_2_. The molecular ion peak at *m/z* 394 was observed for S-PFB_2_


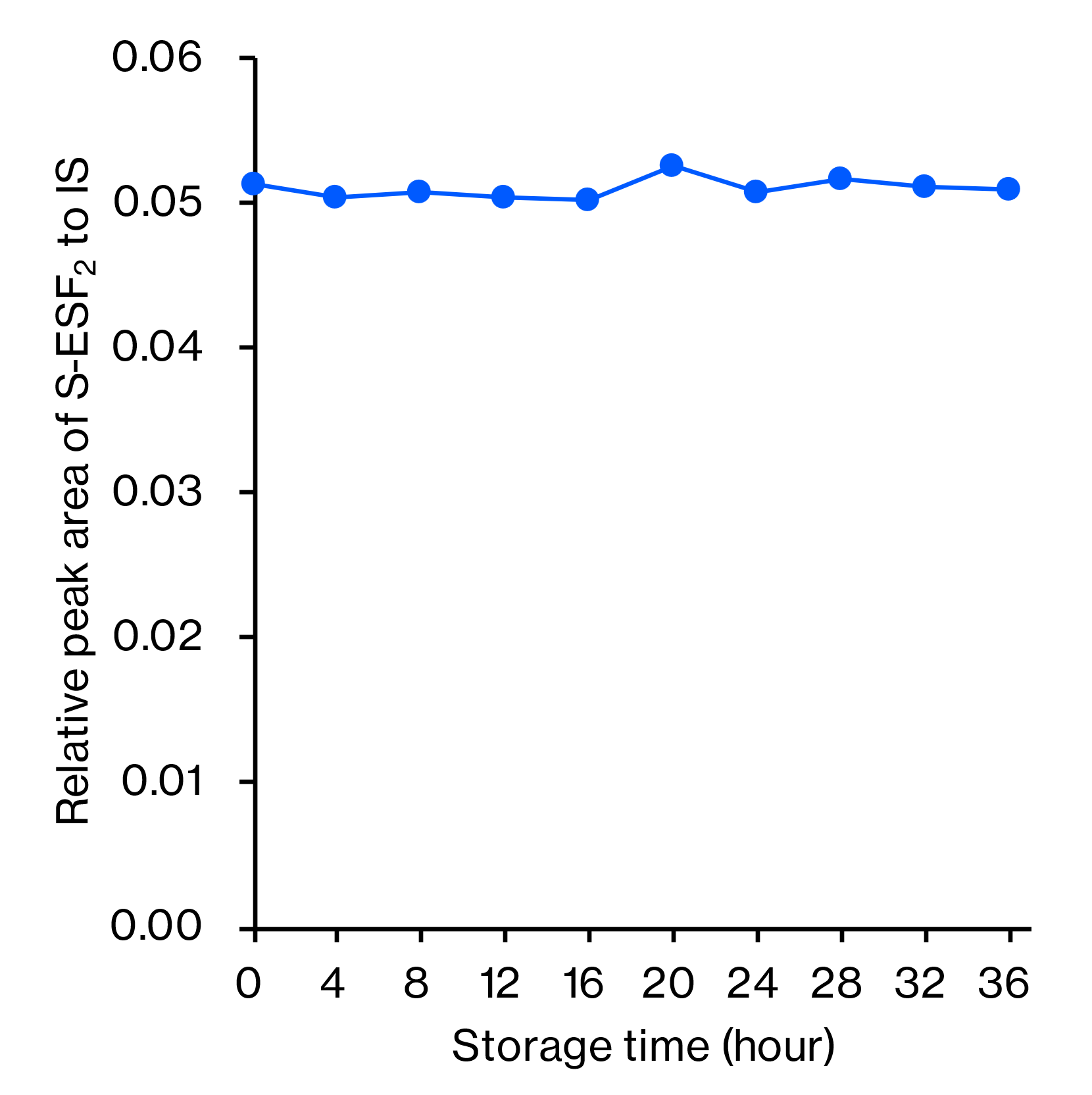


Fig. S3 Effect of storage time of the hexane extract of the derivatized reaction solution on the GC/MS analysis. The extract was stored at room temperature before the analysis

Table S1 Comparison of S-ESF_2_ production in whole blood relative to water (%, *n* = 3)

| Nominal concentration (μg/mL) | Mean ± SD ^a^ |
| --- | --- |
| 0.1 | 77 ± 2.9 |
| 1.0 | 76 ± 6.9 |
| 8.0 | 71 ± 7.5 |

^a^ Standard deviation
